# The Use of Bioaerosol Sampling for Airborne Virus Surveillance in Swine Production Facilities: A Mini Review

**DOI:** 10.3389/fvets.2017.00121

**Published:** 2017-07-27

**Authors:** Benjamin D. Anderson, John A. Lednicky, Montserrat Torremorell, Gregory C. Gray

**Affiliations:** ^1^Division of Infectious Diseases, School of Medicine, Global Health Institute, Duke University, Durham, NC, United States; ^2^Department of Environmental and Global Health, College of Public Health & Health Professions, Emerging Pathogens Institute, University of Florida, Gainesville, FL, United States; ^3^Department of Veterinary Population Medicine, College of Veterinary Medicine, University of Minnesota–Twin Cities, Saint Paul, MN, United States

**Keywords:** bioaerosols, viruses, swine, animal production, zoonoses, air sampling

## Abstract

Modern swine production facilities typically house dense populations of pigs and may harbor a variety of potentially zoonotic viruses that can pass from one pig generation to another and periodically infect human caretakers. Bioaerosol sampling is a common technique that has been used to conduct microbial risk assessments in swine production, and other similar settings, for a number of years. However, much of this work seems to have been focused on the detection of non-viral microbial agents (i.e., bacteria, fungi, endotoxins, etc.), and efforts to detect viral aerosols in pig farms seem sparse. Data generated by such studies would be particularly useful for assessments of virus transmission and ecology. Here, we summarize the results of a literature review conducted to identify published articles related to bioaerosol generation and detection within swine production facilities, with a focus on airborne viruses. We identified 73 scientific reports, published between 1991 and 2017, which were included in this review. Of these, 19 (26.7%) used sampling methodology for the detection of viruses. Our findings show that bioaerosol sampling methodologies in swine production settings have predominately focused on the detection of bacteria and fungi, with no apparent standardization between different approaches. Information, specifically regarding virus aerosol burden in swine production settings, appears to be limited. However, the number of viral aerosol studies has markedly increased in the past 5 years. With the advent of new sampling technologies and improved diagnostics, viral bioaerosol sampling could be a promising way to conduct non-invasive viral surveillance among swine farms.

## Introduction

Bioaerosols can be defined as fine particles ranging in size and composition that are suspended in the air and considered to be derived from a biological source or to affect a biological target (1). Such particles can contain or consist of bacteria, fungi, organic and inorganic particulates, toxins, and viruses. Considerable research has been conducted to understand how bioaerosols impact human health in both indoor and outdoor settings, with a particular emphasis on occupational exposures (2–7). With the intensification of animal production over the past 30 years, and the rise of farms housing large number of animals, bioaerosols have become a particular concern to the health of other animals, workers, and the communities located near such facilities (8). There have been efforts directed at evaluating these bioaerosol risks (9–17), though data related specifically to the detection of airborne viruses still remain largely limited. Most studies have instead focused their sampling strategies on bacteria, fungi, particulates, and endotoxins. This lack of targeted virus sampling is worrisome given that the airborne transmission pathway has been identified as important to the movement of viruses in and between production facilities (18, 19). As such, methodologies that fully assess the risks of bioaerosol exposures in animal production facilities are important for the design and implementation of effective interventions to mitigate the exposure risks to humans and animals in the surrounding environment.

This literature review was conducted to better understand the source and types of viral bioaerosols within and around swine production facilities, the scope of sampling methodologies, and the current state of research. The information summarized from this review may also serve as a collated resource for other researchers.

## Materials and Methods

### Search Strategy and Selection Criteria

Following PRISMA guidelines, a systematic online search of three scientific abstract indexing databases (PubMed, Web of Science, and CAB Abstracts), with no restriction on year of publication, was performed using the following structured query: (bioaerosol* or bio-aerosol*) and (swine or pig* or hog* or barrow* or gilt* or sow or sows or boar or boars or porcine or pork or suidae or sus scrofa). Given that PubMed is a database specific to biomedical and life science research, Web of Science and CAB Abstract databases were included to capture a broader range of disciplines and information sources, including engineering, agriculture, and other technical journals. Search results were manually reviewed and abstracts meeting the following inclusion criteria were retained: (1) peer-reviewed and published scientific report, (2) research occurred in an experimental swine unit, swine production facility, or market, and (3) a bioaerosol sampling strategy was utilized. Articles that were reviews, comments to editor, perspectives, personal opinions, did not present sampling result data, did not have a full-text article available in English, or did not meet the inclusion criteria listed above were excluded. Full reports were reviewed and summarized according to their date of publication.

## Results

### Search Results and Study Selection

From a search conducted April 5, 2017, results yielded 68 publications from PubMed, 180 publications from Web of Science, and 90 publications from CAB Abstracts. After 128 duplicates were removed, 210 publications remained. These were screened, and 114 articles that did not meet the initial inclusion criteria were removed leaving 96 articles. Finally, 23 full-text articles that did not present sufficient data, were not available in English, or did not sufficiently describe the methods were also removed, resulting in a final article count of 73 (Figure S1 in Supplementary Material).

### Study Characteristics

After the selection and screening procedures were completed, 73 scientific reports that met the inclusion criteria for this review remained (Table S1 in Supplementary Material) (19–91). Articles were published between 1991 and 2017 (Figure S2 in Supplementary Material). Only 8 of 73 (11.0%) articles were published in the 1990s (20–27), whereas 40 of 73 (54.8%) have been published since the beginning of 2010 (52–91). Of the 73 reviewed articles, 41 (56.2%) included methodologies to evaluate bacteria, 25 (34.2%) to evaluate fungi, 19 (26.0%) to evaluate viruses, 16 (21.9%) to evaluate dust and particulates, 11 (15.1%) to evaluate endotoxins, 8 (11.0%) to evaluate gases, 6 (8.2%) to evaluate 16s rRNA genes, 3 (4.1%) each to evaluate antibiotic resistance and organic compounds, and 1 (1.4%) each to evaluate archaea and chemical markers (Table S2 in Supplementary Material). Studies were conducted in the United States (*n* = 35), Canada (*n* = 9), Republic of Korea (*n* = 8), China (*n* = 4), Denmark (*n* = 4), Poland (*n* = 4), Germany (*n* = 3), Australia (*n* = 2), The Netherlands (*n* = 2), Belgium (*n* = 1), Portugal (*n* = 1), Sweden (*n* = 1), Switzerland (*n* = 1), and the United Kingdom (*n* = 1). Primary bioaerosol sampling methodologies utilized across the studies included a single, two- and six-stage Andersen sampler, all-glass impingers, button sampler, various filter collection cassettes in combination with molecular sequencing and mass spectrometry, slit sampler, and liquid cyclonic collector.

## Discussion

In this literature review, articles were searched and summarized to better understand the source and types of bioaerosols detected in and around swine production facilities, as well as the sampling methods used to detect them. Based on the articles evaluated, different bacteria were the predominantly sampled bioaerosol targets, with several studies identifying elevated levels of both Gram-positive and Gram-negative bacteria, including *Actinobacillus pleuropneumoniae, Escherichia coli, Staphylococcus aureus*, and *Streptococcus suis*, among others. Antibiotic resistance among detected bacterial pathogens was also assessed in several studies, which found a high rate of antibiotic-resistant bacteria both within and downwind from swine production facilities (35, 40, 81). Gibbs et al. found that antibiotic-resistant microbes were detectable at least 150 m downwind from the sampled swine barn at concentrations that could pose a threat to individuals working in the barns, as well as those living within close proximity (40). Arfken et al. found that fecal spray fields using swine feces as fertilizer could be a source of aerosolization and introduction of antibiotic-resistant bacteria into the environment (81).

Seasonal variation was assessed in multiple studies identifying trends in the rates of bioaerosol detection between seasons, which also varied based on the type of target being sampled (21, 44, 46, 51, 77). Kim et al. found that fungal spores were more likely to be detected during the warmer summer months, whereas bacteria and particulates were found at higher concentrations during the winter (44). Anderson et al. compared detections of influenza A virus (IAV) RNA between summer and winter, finding an association between an increased detection rate during the winter and temperatures below 20°C (85).

One of the first studies to assess bioaerosol transmission of viruses between swine was conducted in 1997 by Torremorell et al., which evaluated the airborne transmission of porcine reproductive and respiratory syndrome virus (PRRSV) under experimental conditions among nursery pigs (25). In this study, the authors documented short-distance airborne transmission of PRRSV and found that transmission was strain dependent. Otake et al. then conducted a study in 2002 which also evaluated the propensity of PRRSV to be transmitted by aerosol, but this time under field conditions (19). Results demonstrated that naïve pigs with direct or indirect contact with PRRSV-infected pigs also became infected, while sentinel pigs placed 1 and 30 m away from exhaust fans of the barn containing the infected pigs did not. Four years later in 2006, Hermann et al. carried out an optimization study using an all-glass impinger for the detection of PRRSV and IAV under experimental conditions (92). The study ultimately concluded that given the lack of standard methodology for isolating certain pathogens in aerosols, future methods should be individually optimized and validated for each pathogen of interest.

These seminal works were followed by 16 additional studies (47, 50, 55, 56, 61, 63–65, 71, 74, 75, 80, 82, 85, 87, 88, 90) between 2009 and 2017, which found detectable levels of viral RNA from aerosolized PRRSV, IAV, porcine epidemic diarrhea virus (PEDV), and porcine circovirus type 2 (PCV2) (Table 1). These studies were either experimental or field based in their design and varied greatly in their research objectives, including the evaluation of collection efficiencies for different sampling devices, estimating the burden of aerosolized viruses, and evaluating the efficiency of air cleaning systems or other similar interventions aimed at reducing bioaerosols in pig facilities. Notably, the experimental studies appeared to benefit greatly from inclusion of more robust controls in their assessment, while the field-based studies seemed to be able to better address the questions of transmission risk associated with bioaerosol generation in actual production farms. Given the lack of standardized methodology and the diversity in study designs, it is difficult to make direct comparisons between studies. Such standardization would be essential to verify the consistency and reproducibility of bioaerosol detection results across studies. Furthermore, it would be useful for future aerosol detection studies to incorporate more comprehensive epidemiological approaches to better ascertain the association between human, animal, and environmental risk factors with aerosolization of target viruses. These epidemiological approaches should include larger sample sizes, appropriate controls, and a prospective sampling strategy.

**Table 1:** Evaluation of reviewed bioaerosol studies assessing viruses (*n* = 19).

Reference	Target virus(es)	Strength(s)
Torremorell et al. (25)	Porcine reproductive and respiratory syndrome virus (PRRSV)	Evaluated virus viability Confirmation of source population infection using virus isolation and serology Assessed strain differences Robust controls
Otake et al. (19)	PRRSV	Evaluated virus viability Confirmation of source population infection using virus isolation and serology Assessed strain differences Robust controls Evaluated long-distance transport
Pitkin et al. (50)	PRRSV	Evaluated virus viability Confirmation of source population infection Robust sampling strategy Year-long sampling
Dee et al. (47)	PRRSV	Evaluated virus viability Confirmation of source population infection Robust controls Evaluated long-distance transport
Otake et al. (55)	PRRSV	Evaluated virus viability Confirmation of source population infection Robust controls Evaluated long-distance transport
Verreault et al. (56)	Porcine circovirus type 2 (PCV2)	Multi-year sampling Sensitivity of detection assay explored
Linhares et al. (61)	PRRSV	Evaluated virus viability Confirmation of source population infection Robust controls Pigs sampled concomitantly with air
Corzo et al. (63)	Influenza A virus (IAV)	Evaluated virus viability Confirmation of source population infection Subtyping conducted Evaluated long-distance transport
Corzo et al. (64)	IAV	Bioaerosol detection and viral secretion in pigs directly compared
de Evgrafov et al. (65)	PCV2	Used controls to rule out contamination Used advanced genomic methods
Alonso et al. (71)	Porcine epidemic diarrhea virus (PEDV)	Evaluated virus viability Confirmation of source population infection Evaluated long-distance transport
Brito et al. (74)	PRRSV	Used controls to rule out contamination Used GIS modeling to correlate sampling with farm density Used sequencing techniques and phylogenetic analysis
Corzo et al. (75)	IAV	Evaluated virus viability Confirmation of source population infection Robust controls Viral shedding assessed over time
Alonso et al. (80)	IAV PRRSV PEDV	Multiple viruses concomitantly assessed Particle size evaluated Evaluated virus viability Confirmation of source population infection Robust controls Pigs sampled concomitantly with air Infectivity of air samples assessed using swine bioassay
Choi et al. (82)	IAV	Human, animal, and environmental sampling conducted concomitantly Evaluated virus viability Documented possible aerosol transmission of swine-sourced virus to humans Sequencing used to compare detected virus RNA gene segments
Anderson et al. (85)	IAV	Human, animal, and environmental sampling conducted concomitantly Seasonal comparisons made Risk factors evaluated Sampling types statistically compared
Neira et al. (87)	IAV	Sampling captured during outbreaks under field settings Animal, environmental, and air sampling conducted concomitantly Evaluated virus viability Sampling types statistically compared
O’Brien and Nonnenmann (88)	IAV	Human exposure directly assessed Two samplers compared Confirmation of source population infection Physical conditions of farms assessed
Alonso et al. (90)	PRRSV PEDV	Particle size evaluated Confirmation of source population infection Sampling types statistically compared

**Figure 1:**
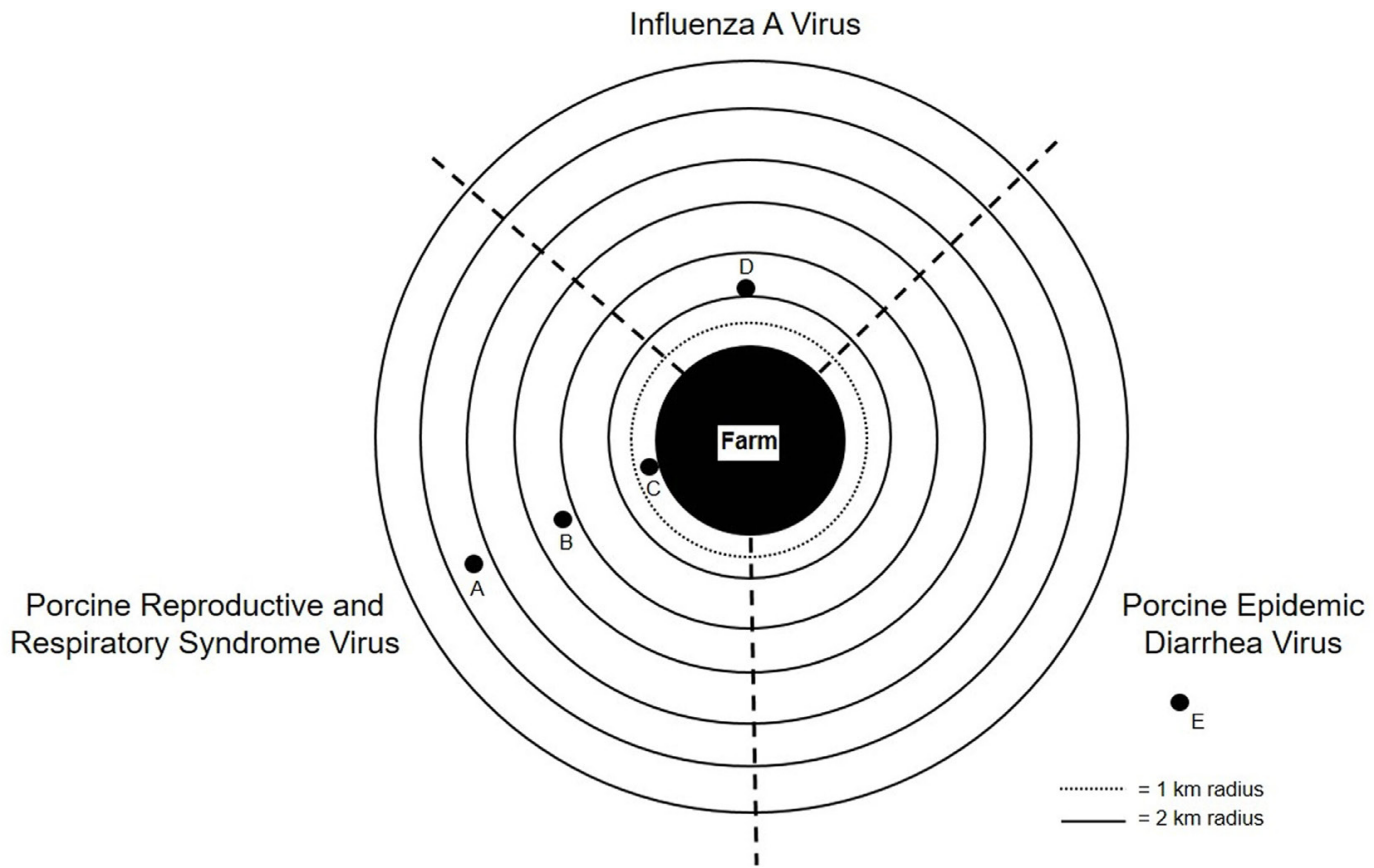
Graphical depiction of influenza A virus (IAV), porcine reproductive and respiratory syndrome virus (PRRSV), and porcine epidemic diarrhea virus (PEDV) RNA detection downwind from farms with infected source populations: (A) PRRSV RNA detected up to 9.1 km away from infected source population; (B) PRRSV RNA detected 4.7 km away from infected source population; (C) PRRSV infects naïve pigs 120 m away from infected source population; (D) IAV RNA detected up to 2.1 km away from infected source population; and (E) PEDV RNA detected up to 16.1 km away from infected source population.

Several studies have documented detection of PRRSV, IAV, and PEDV virus genomic RNAs at various distances downwind from swine barns with infected source populations (47, 50, 55, 63, 71). PEDV RNA was detected up to 16.1 km (71) away, PRRSV RNA up to 9.1 km (47, 50, 55) away, and IAV up to 2.1 km (75) downwind from infected source populations (Figure 1). Furthermore, PRRSV was shown to be infectious 120 m and 4.7 km away from an infected herd (47, 50). These findings suggest that viral bioaerosols could pose a potential risk to other farms and their livestock, or communities located within close proximity to barns with infected pigs, which may undermine current biosecurity practices.

Despite the increase in the number of studies that have been conducted using bioaerosol sampling for the detection of viruses, such approaches may have some limitations given the difficulty of determining the correct sampling parameters (i.e., flow rate, collection media, volume, etc.). Optimization is essential to capture aerosolized viruses and to maintain their viability throughout the collection process. Identifying viable virus is important because it allows for a more informed risk assessment in the environments being sampled. A viable virus carries a greater transmission risk to exposed humans or animals. In contrast, if the virus is already inactivated (by UV light or drying or other means) when it is detected, it likely poses minimal risk in terms of airborne transmission. This emphasizes the need for additional field-based studies that focus on the optimization of collection parameters to improve the recovery of viable virus.

Another important factor that contributes to the viability of aerosolized viruses is particle size. In a study conducted by Zuo et al., the authors demonstrated a close relationship between particle size of three different animal viruses (gastroenteritis virus, swine IAV, and avian IAV) and the infectivity and survivability of those viruses after collection using a bioaerosol sampling device (93). Though there were some differences between the three viruses tested, in general it was shown that the larger particle sizes (300–450 nm) had a higher survivability compared to particles measuring closer to the actual size of the virions (100–200 nm). Similarly, Alonso et al. found in their 2015 study that the viability of IAV and PRRSV was particle size dependent, only being able to isolate viable virus from particles larger than 2.1 µm (80). Given this preliminary association, which is still not fully defined, future viral bioaerosol sampling studies would benefit from the routine inclusion of methodology to evaluate particle size as it relates to viability. Only a few studies in this review incorporated such an approach, however, this is likely due to limitations in the detection technology.

A further challenge to using bioaerosol sampling as a method for virus surveillance and risk assessment is that commercially available air samplers are not optimally designed for the collection of submicron particles (<1 μm), for which the collection efficiencies tend to be low. Samplers are instead designed for the collection of micrometer-sized particles such as fungal spores and bacteria (94, 95). Using an experimental model, Hogan et al. evaluated collection efficiencies for three bioaerosol samplers (AGI-30, the SKC BioSampler and a frit bubbler), and found the collection efficiency for bacteriophages MS2 (*d* = 27.5 nm) and T3 (*d* = 45 nm) to be extremely low (5–10%) (96). Though conducted under a controlled setting, these findings indicate the collection efficiency for viruses to be markedly lower compared to that of larger bacteria and fungi in aerosols, which are often recovered at close to 100% efficiency. Despite these limitations, new optimization studies of different bioaerosol sampling devices have shown some promise in overcoming the challenge of low detection efficiency for some swine viruses (63, 64, 75, 82). Noteworthy, is a recent study conducted by Pan et al. which demonstrated a 93% recovery of aerosolized bacteriophage MS2 (2–5 µm in diameter) using a novel growth tube collector (GTC) (97). A subsequent study conducted by Lednicky et al. evaluated the same GTC sampling device with IAV, and demonstrated an 84% collection efficiency (98). These studies highlight how improvements in the sampling technology may continue to increase the sensitivity of viral bioaerosol sampling.

Overall, the bioaerosol sampling research studies evaluated in this review were predominately focused on the detection of bacteria and fungi and relied on broad-spectrum microbial detection as an indicator for overall bioaerosol burden. In addition, sampling strategies were found to utilize a wide variety of methodologies, with no apparent consistency between research groups, suggesting a lack of standard methods for performing bioaerosol studies in swine production settings. Many of the researchers in the reviewed studies agreed that there are insufficient data regarding virus aerosol burdens in swine production facilities to assess their potential impact upon human and animal health. Furthermore, only a few studies have since been conducted using multi-faceted strategies to sample animals, humans, and the environment. This approach will be critical in future studies to better understand transmission pathways and virus ecology. Finally, only one study was conducted in Mainland China, a region with the largest and fastest growing swine industry in the world (85).

Recent breaches in animal production biosecurity in the United States, resulting in the incursion of H5N2 avian IAV (99, 100), as well as outbreaks of H3N2v IAV of swine origin in swine shows (101) and experimental challenge studies (75), suggest that virus aerosol transmission may play a much larger role in zoonotic disease transmission between pigs and from pigs to people. Infectious diseases, which affect the swine production industry, particularly PRRSV, may be further exacerbated by the dissemination of aerosolized viruses. As such, the further development and optimization of bioaerosol sampling technology seems prudent, as this review suggests bioaerosol sampling is a promising way to conduct non-invasive viral surveillance among swine farms and perhaps other, similar ecological settings.

## Author Contributions

BA conducted the literature review and wrote the manuscript; JL and MT helped revise the manuscript to add important scientific content and refine the interpretation of the results; GG conceived of the idea of the review and helped revise the manuscript to add important scientific content and refine the interpretation of the results. All the authors reviewed the final version of the manuscript and agreed to its submission.

## Conflict of Interest Statement

The authors declare that the research was conducted in the absence of any commercial or financial relationships that could be construed as a potential conflict of interest.
