# Supplementary material for: The Use of Bioaerosol Sampling for Airborne Virus Surveillance in Swine Production Facilities: A Mini Review
Source: Front Vet Sci. 2017 Jul 27;4:121. doi: 10.3389/fvets.2017.00121 (PMC5529434; doi:10.3389/fvets.2017.00121)
Supplement: Supplementary file 1 [file Data_Sheet_1.DOCX]

**The Use of Bioaerosol Sampling for Airborne Virus Surveillance
in Swine Production Facilities: A Mini Review**

Benjamin D. Anderson*, John A. Lednicky, Montserrat Torremorell, and Gregory C. Gray

***Correspondence:** Benjamin D. Anderson, MPH, PhD, CPH, [benjamin.anderson2@duke.edu](mailto:benjamin.anderson2@duke.edu)

**1 Supplementary Tables**

**1.1 Supplementary Table 1**

| Table S1. Summary of bioaerosol studies conducted in or around swine farms between 1991 and 2017 (N=73). | | | |
| --- | --- | --- | --- |
| **Publication** | **Year** | **Sampler(s)** | **Sampling Target(s)** |
| Butera et al. (1) | 1991 | Six-Stage Viable Andersen Cascade Impactor | Fungi  Bacteria  Dust |
| Thorne et al. (2) | 1992 | Six-Stage Viable Andersen Cascade Impactor  All-glass Impinger (AGI-30)  Nuclepore Filtration-Elution | Bacteria  Enteric bacteria  Total fungi |
| Schwartz et al. (3) | 1995 | Filtration | Dust |
| Lange et al. (4) | 1997 | All-glass Impinger (AGI-30)  May 3-stage Impinger | Bacteria |
| Thorne et al. (5) | 1997 | Filtration | Endotoxins |
| Torremorell et al. (6) | 1997 | All-glass Impinger (AGI-30) | Virus  Bacteria |
| Mackiewicz (7) | 1998 | Selective Aerobioscope  Filtration | Bacteria  Fungi  Dust |
| Seedorf et al. (8) | 1998 | Automated Bioaerosol Sampler with Agar Plates | Endotoxins  Bacteria  Enteric bacteria  Fungi |
| Duchaine et al. (9) | 2001 | All-glass Impinger  Filtration | Endotoxins |
| Martens et al. (10) | 2001 | All-glass Impinger (AGI-30)  Filtration | Endotoxins  Bacteria  Fungi  Organic Compounds  Gases |
| Predicala et al. (11) | 2001 | Filtration | Carbon dioxide  Ammonia  Dust |
| Szponar (12) | 2001 | Filtration | Chemical markers  Dust |
| Otake et al. (13) | 2002 | All-glass Impinger (AGI-30) | Virus |
| Predicala et al. (14) | 2002 | Filtration  Six-Stage Viable Andersen Cascade Impactor | Bacteria |
| Adhikari et al. (15) | 2004 | Button Aerosol Sampler | Fungi |
| Agranovski et al. (16) | 2004 | Ultraviolet Aerodynamic Particle Sizer  Six-Stage Viable Andersen Cascade Impactor  All-glass Impinger (AGI-30) | Bacteria  Fungi |
| Gibbs et al. (17) | 2004 | Two-Stage Viable Andersen Cascade Impactor | Bacteria  Antibiotic resistance |
| Chi (18) | 2005 | All-glass Impinger (AGI-30) | Bacteria  Fungi |
| Chinivasagam (19) | 2005 | Six-Stage Viable Andersen Cascade Impactor  All-glass Impinger (AGI-30) | Bacteria |
| Jo (20) | 2005 | Single-Stage Viable Andersen Cascade Impactor | Bacteria  Fungi |
| Karwowska (21) | 2005 | MAS-100 Impactor | Bacteria  Fungi |
| Gibbs et al. (22) | 2006 | Two-Stage Viable Andersen Cascade Impactor | Bacteria  Antibiotic resistance |
| Green et al. (23) | 2006 | Two-Stage Viable Andersen Cascade Impactor | Bacteria |
|  |  |  |  |
| Lee et al. (24) | 2006 | Filtration | Bacteria  Fungi  Dust/Particulates |
| Kim et al. (25) | 2007 | Single-Stage Viable Andersen Cascade Impactor  All-glass Impinger (AGI-30)  Filtration | Bacteria  Fungi  Dust/Particulates  Gases |
| Kim et al. (26) | 2007 | All-glass Impinger (AGI-30)  Filtration | Bacteria  Fungi  Dust/Particulates  Gases |
| Ko et al. (27) | 2008 | All-glass Impinger (AGI-30) | Bacteria  Fungi |
| Nehme et al. (28) | 2008 | All-glass Impinger (AGI-30) | 16s rRNA gene |
| Dee et al. (29) | 2009 | Liquid Cyclonic Collector | Virus  Bacteria |
| Lavoie et al. (30) | 2009 | Filtration | Endotoxins  Bacteria  Dust |
| Nehme et al. (31) | 2009 | Filter Cassettes | Archaea |
| Pitkin et al. (32) | 2009 | Liquid Cyclonic Collector | Virus |
| Thorne et al. (33) | 2009 | All-glass Impinger (AGI-30)  Filtration | Endotoxins  Bacteria  Fungi  Dust/Particulates  Gases |
| Ko et al. (34) | 2010 | All-glass Impinger (AGI-30)  Filtration | Endotoxins  Bacteria  Fungi |
| Letourneau et al. (35) | 2010 | Six-Stage Viable Andersen Cascade Impactor  All-glass Impinger (AGI-30) | Bacteria |
| Letourneau et al. (36) | 2010 | Six-Stage Viable Andersen Cascade Impactor  All-glass Impinger (AGI-30)  Filtration | Endotoxins  Bacteria  Fungi  Dust |
| Otake et al. (37) | 2010 | Liquid Cyclonic Collector | Virus  Bacteria |
| Verreault et al. (38) | 2010 | Filtration | Virus  Bacteria  Dust/Particulates |
| Keessen et al. (39) | 2011 | MicroBio MB1 Bioaerosol Impactor | Bacteria |
| Cyprowski et al. (40) | 2012 | Filtration | Dust/Particulates  Organic Compounds |
| Hong et al. (41) | 2012 | Filtration | 16s rRNA gene |
| Kristiansen et al. (42) | 2012 | Filtration | Bacteria  Fungi |
| Linhares et al. (43) | 2012 | Liquid Cyclonic Collector | Virus |
| Sowiak et al. (44) | 2012 | Filtration | Bacteria  Fungi  Dust/Particulates |
| Corzo et al. (45) | 2013 | Liquid Cyclonic Collector | Virus |
| Corzo et al. (46) | 2013 | Liquid Cyclonic Collector | Virus |
| De Evgrafov et al. (47) | 2013 | Swirling Liquid All-glass Impinger (SKC and Omni 3000) | Virus  16s rRNA gene |
| Masclaux et al. (48) | 2013 | MAS-100 Eco Impactor  Filtration | Endotoxins  Bacteria  Fungi |
| Pavilonis et al. (49) | 2013 | Absorbent Cartridge | Gases |
| Viegas et al. (50) | 2013 | Impaction on Agar | Fungi |
| Yang et al. (51) | 2013 | Filtration | Endotoxins  Fungi  Particulates |
| Agerso et al. (52) | 2014 | Filtration | Bacteria |
| Alonso et al. (53) | 2014 | Liquid Cyclonic Collector | Virus |
| Andersen et al. (54) | 2014 | Filtration | Organic Compounds  Gases |
| Bonifait et al. (55) | 2014 | Liquid Cyclonic Collector | Bacteria |
| Brito et al. (56) | 2014 | Liquid Cyclonic Collector | Virus |
| Corzo et al. (57) | 2014 | Liquid Cyclonic Collector | Virus |
| Damte et al. (58) | 2014 | Filtration | Bacteria |
| Kumari (59) | 2014 | Filtration | 16s rRNA gene |
| Lee (60) | 2014 | Filtration | Fungi |
| Van Ransbeeck et al. (61) | 2014 | Spectrometer  Infrared Photo Acoustic Detector | Particulates  Gases |
| Alonso et al. (62) | 2015 | Viable Andersen Cascade Impactor  Liquid Cyclonic Collector | Virus |
| Arken et al. (63) | 2015 | Filtration | Bacteria  16s rRNA gene |
| Choi et al. (64) | 2015 | Liquid Cyclonic Collector | Virus |
| Kumari et al. (65) | 2015 | Filtration | Bacteria  16s rRNA gene |
| Wang et al. (66) | 2015 | Button Aerosol Sampler  IOM Inhalable Dust Sampler  NIOSH Personal Bioaerosol Cyclone Sampler  37-mm Filter Cassette Sampler | Bacteria  Fungi |
| Anderson et al. (67) | 2016 | Swirling Liquid All-glass Impinger (SKC) | Virus |
| Ferguson et al. (68) | 2016 | Viable Andersen Cascade Impactor | Bacteria |
| Neira et al. (69) | 2016 | Liquid Cyclonic Collector | Virus |
| O’Brien (70) | 2016 | NIOSH Personal Bioaerosol Cyclone Sampler  Filtration | Virus |
| Roque et al. (71) | 2016 | Biostage-1 Cascade Impactor | Endotoxins  Bacteria  Fungi |
| Alonso et al. (72) | 2017 | Viable Andersen Cascade Impactor  Tisch Cascade Impactor | Virus |
| Walser et al. (73) | 2017 | Liquid Cyclonic Collector | Bacteria |

**1.2 Supplementary Table 2**

| Table S2. Summary of selected articles by sampling target (n=73). | | |
| --- | --- | --- |
| **Sampling Target** | **No. (%)** | **References** |
| Bacteria | 41 (56.2) | (1, 2, 4, 6-8, 10, 14, 16-27, 29, 30, 33-39, 42, 44, 48, 52, 55, 58, 63, 65, 66, 68, 71, 73) |
| Fungi | 25 (34.2) | (1, 2, 7, 8, 10, 15, 16, 18, 20, 21, 24-27, 33, 34, 36, 42, 44, 48, 50, 51, 60, 66, 71) |
| Virus | 19 (26.0) | (6, 13, 29, 32, 37, 38, 43, 45-47, 53, 56, 57, 62, 64, 67, 69, 70, 72) |
| Dust/Particulates | 16 (21.9) | (1, 3, 7, 11, 12, 24-26, 30, 33, 36, 38, 40, 44, 51, 61) |
| Endotoxin | 11 (15.1) | (5, 8-10, 30, 33, 34, 36, 48, 51, 71) |
| Gases | 8 (11.0) | (10, 11, 25, 26, 33, 49, 54, 61) |
| 16s rRNA gene | 6 (8.2) | (28, 41, 47, 59, 63, 65) |
| Antibiotic resistance | 3 (4.1) | (17, 22, 63) |
| Organic compounds | 3 (4.1) | (10, 40, 54) |
| Archaea | 1 (1.4) | (31) |
| Chemical markers | 1 (1.4) | (12) |

**2 Supplementary Figures**

**2.1 Supplementary Figure 1**

**
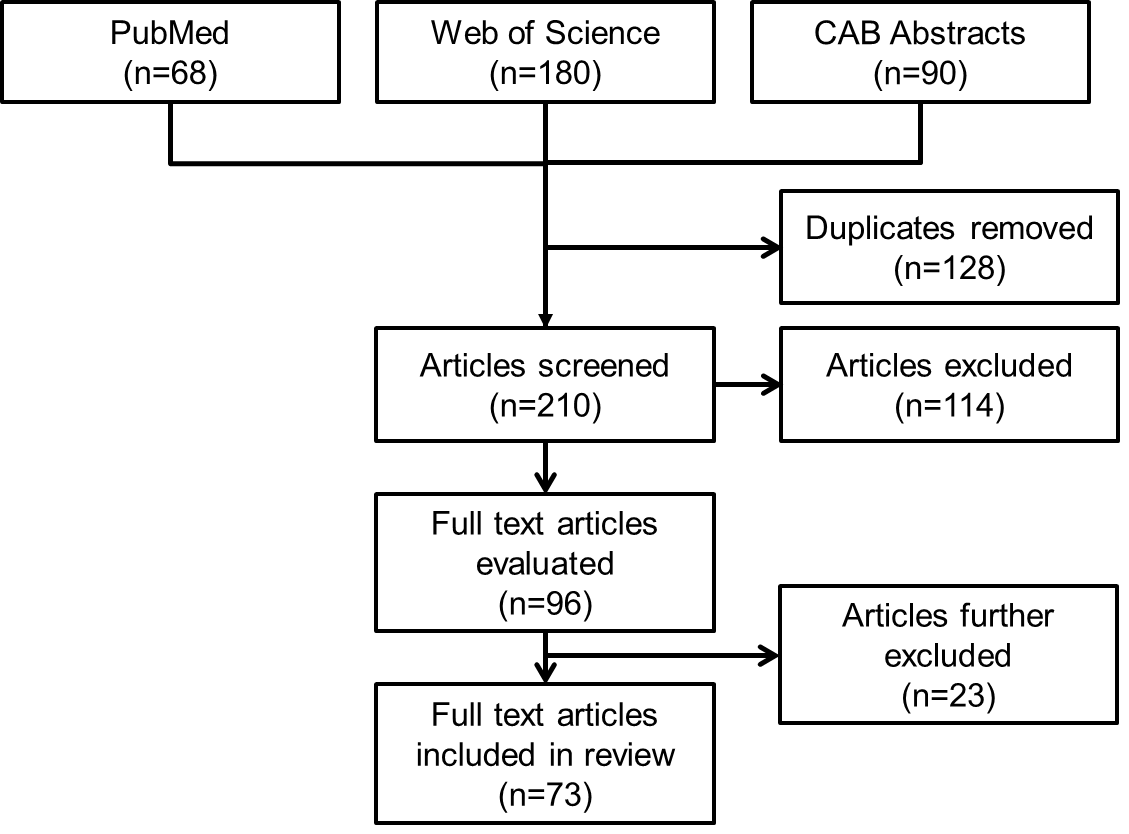
**

Figure S1. Article selection in the literature review.

**2.2 Supplementary Figure 2**

**
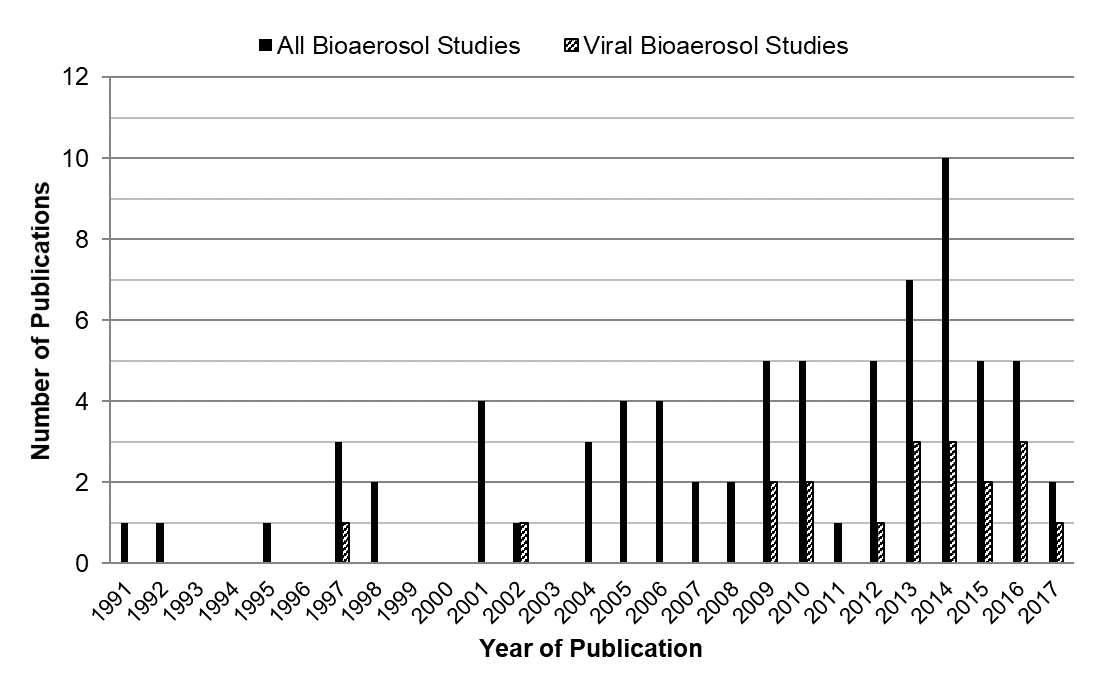
**

Figure S2. Number of publications by year and type meeting the inclusion criteria from 1991-2017.

**Supplementary References**

1. Butera M, Smith JH, Morrison WD, Hacker RR, Kains FA, Ogilvie JR. Concentration of Respirable Dust and Bioaerosols and Identification of Certain Microbrial Types in a Hog-Growing Facility. Canadian Journal of Animal Science. 1991;71(2):271-7.

2. Thorne PS, Kiekhaefer MS, Whitten P, Donham KJ. Comparison of Bioaerosol Sampling Methods in Barns Housing Swine. Applied and Environmental Microbiology. 1992;58(8):2543-51.

3. Schwartz DA, Donham KJ, Olenchock SA, Popendorf WJ, Vanfossen DS, Burmeister LF, et al. Determinants of Longitudinal Changes in Spirometric Function Among Swine Confinement Operators and Farmers. American Journal of Respiratory and Critical Care Medicine. 1995;151(1):47-53.

4. Lange JL, Thorne PS, Lynch N. Application of flow cytometry and fluorescent in situ hybridization for assessment of exposures to airborne bacteria. Appl Environ Microbiol. 1997;63(4):1557-63.

5. Thorne PS, Reynolds SJ, Milton DK, Bloebaum PD, Zhang X, Whitten P, et al. Field evaluation of endotoxin air sampling assay methods. Am Ind Hyg Assoc J. 1997;58(11):792-9.

6. Torremorell M, Pijoan C, Janni K, Walker R, Joo HS. Airborne transmission of Actinobacillus pleuropneumoniae and porcine reproductive and respiratory syndrome virus in nursery pigs. Am J Vet Res. 1997;58(8):828-32.

7. Mackiewicz B. Study on Exposure of Pig Farm Workers to Bioaerosols, Immunologic Reactivity and Health Effects. Annals of Agricultural and Environmental Medicine. 1998;5(2):169-75.

8. Seedorf J, Hartung J, Schroder M, Linkert KH, Phillips VR, Holden MR, et al. Concentrations and emissions of airborne endotoxins and microorganisms in livestock buildings in Northern Europe. Journal of Agricultural Engineering Research. 1998;70(1):97-109.

9. Duchaine C, Thorne PS, Meriaux A, Grimard Y, Whitten P, Cormier Y. Comparison of endotoxin exposure assessment by bioaerosol impinger and filter-sampling methods. Appl Environ Microbiol. 2001;67(6):2775-80.

10. Martens W, Martinec M, Zapirain R, Stark M, Hartung E, Palmgren U. Reduction potential of microbial, odour and ammonia emissions from a pig facility by biofilters. Int J Hyg Environ Health. 2001;203(4):335-45.

11. Predicala BZ, Maghirang RG, Jerez SB, Urban JE, Goodband RD. Dust and bioaerosol concentrations in two swine-finishing buildings in Kansas. Transactions of the Asabe. 2001;44(5):1291-8.

12. Szponar B, Larsson L. Use of mass spectrometry for characterising microbial communities in bioaerosols. Annals of Agricultural and Environmental Medicine. 2001;8(2):111-7.

13. Otake S, Dee SA, Jacobson L, Torremorell M, Pijoan C. Evaluation of aerosol transmission of porcine reproductive and respiratory syndrome virus under controlled field conditions. Vet Rec. 2002;150(26):804-8.

14. Predicala BZ, Urban JE, Maghirang RG, Jerez SB, Goodband RD. Assessment of bioaerosols in swine barns by filtration and impaction. Current Microbiology. 2002;44(2):136-40.

15. Adhikari A, Reponen T, Lee SA, Grinshpun SA. Assessment of human exposure to airborne fungi in agricultural confinements: Personal inhalable sampling versus stationary sampling. Annals of Agricultural and Environmental Medicine. 2004;11(2):269-77.

16. Agranovski V, Ristovski Z, Blackall PJ, Morawska L. Size-selective assessment of airborne particles in swine confinement building with the UVAPS. Atmospheric Environment. 2004;38(23):3893-901.

17. Gibbs SG, Green CF, Tarwater PM, Scarpino PV. Airborne antibiotic resistant and nonresistant bacteria and fungi recovered from two swine herd confined animal feeding operations. Journal of Occupational and Environmental Hygiene. 2004;1(11):699-706.

18. Chi MC, Li CS. Fluorochrome and fluorescent in situ hybridization to monitor bioaerosols in swine buildings. Aerosol Science and Technology. 2005;39(11):1101-10.

19. Chinivasagam HN, Blackall PJ. Investigation and application of methods for enumerating heterotrophs and Escherichia coli in the air within piggery sheds. Journal of Applied Microbiology. 2005;98(5):1137-45.

20. Jo W-K, Kang J-H. Exposure levels of airborne bacteria and fungi in Korean swine and poultry sheds. Archives of Environmental & Occupational Health. 2005;60(3):140-6.

21. Karwowska E. Microbiological air contamination in farming environment. Polish Journal of Environmental Studies. 2005;14(4):445-9.

22. Gibbs SG, Green CF, Tarwater PM, Mota LC, Mena KD, Scarpino PV. Isolation of antibiotic-resistant bacteria from the air plume downwind of a swine confined or concentrated animal feeding operation. Environ Health Perspect. 2006;114(7):1032-7.

23. Green CF, Gibbs SG, Tarwater PM, Mota LC, Scarpino PV. Bacterial plume emanating from the air surrounding swine confinement operations. Journal of Occupational and Environmental Hygiene. 2006;3(1):9-15.

24. Lee SA, Adhikari A, Grinshpun SA, McKay R, Shukla R, Reponen T. Personal exposure to airborne dust and microorganisms in agricultural environments. Journal of Occupational and Environmental Hygiene. 2006;3(3):118-30.

25. Kim KY, Ko HJ, Kim HT, Kim YS, Roh YM, Lee CM, et al. Monitoring of aerial pollutants emitted from swine houses in Korea. Environmental Monitoring and Assessment. 2007;133(1-3):255-66.

26. Kim KY, Ko HJ, Kim HT, Kim YS, Roh YM, Lee CM, et al. Influence of extreme seasons on airborne pollutant levels in a pig-confinement building. Archives of Environmental & Occupational Health. 2007;62(1):27-32.

27. Ko G, Simmons OD, III, Likirdopulos CA, Worley-Davis L, Williams M, Sobsey MD. Investigation of Bioaerosols Released from Swine Farms using Conventional and Alternative Waste Treatment and Management Technologies. Environmental Science & Technology. 2008;42(23):8849-57.

28. Nehme B, Letourneau V, Forster RJ, Veillette M, Duchaine C. Culture-independent approach of the bacterial bioaerosol diversity in the standard swine confinement buildings, and assessment of the seasonal effect. Environmental Microbiology. 2008;10(3):665-75.

29. Dee S, Otake S, Oliveira S, Deen J. Evidence of long distance airborne transport of porcine reproductive and respiratory syndrome virus and Mycoplasma hyopneumoniae. Vet Res. 2009;40(4):39.

30. Lavoie J, Godbout S, Lemay SP, Belzile M. Impact of in-barn manure separation on biological air quality in an experimental setup identical to that in swine buildings. J Agric Saf Health. 2009;15(3):225-40.

31. Nehme B, Gilbert Y, Letourneau V, Forster RJ, Veillette M, Villemur R, et al. Culture-Independent Characterization of Archaeal Biodiversity in Swine Confinement Building Bioaerosols. Applied and Environmental Microbiology. 2009;75(17):5445-50.

32. Pitkin A, Deen J, Dee S. Use of a production region model to assess the airborne spread of porcine reproductive and respiratory syndrome virus. Veterinary microbiology. 2009;136(1-2):1-7.

33. Thorne PS, Ansley AC, Perry SS. Concentrations of Bioaerosols, Odors, and Hydrogen Sulfide Inside and Downwind from Two Types of Swine Livestock Operations. Journal of Occupational and Environmental Hygiene. 2009;6(4):211-20.

34. Ko G, Simmons OD, III, Likirdopulos CA, Worley-Davis L, Williams CM, Sobsey MD. Endotoxin Levels at Swine Farms Using Different Waste Treatment and Management Technologies. Environmental Science & Technology. 2010;44(9):3442-8.

35. Letourneau V, Nehme B, Meriaux A, Masse D, Cormier Y, Duchaine C. Human pathogens and tetracycline-resistant bacteria in bioaerosols of swine confinement buildings and in nasal flora of hog producers. International Journal of Hygiene and Environmental Health. 2010;213(6):444-9.

36. Letourneau V, Nehme B, Meriaux A, Masse D, Duchaine C. Impact of production systems on swine confinement buildings bioaerosols. J Occup Environ Hyg. 2010;7(2):94-102.

37. Otake S, Dee S, Corzo C, Oliveira S, Deen J. Long-distance airborne transport of infectious PRRSV and Mycoplasma hyopneumoniae from a swine population infected with multiple viral variants. Veterinary microbiology. 2010;145(3-4):198-208.

38. Verreault D, Letourneau V, Gendron L, Masse D, Gagnon CA, Duchaine C. Airborne porcine porcine circovirus in Canadian swine confinement buildings. Veterinary microbiology. 2010;141(3-4):224-30.

39. Keessen EC, Donswijk CJ, Hol SP, Hermanus C, Kuijper EJ, Lipman LJA. Aerial dissemination of Clostridium difficile on a pig farm and its environment. Environ Res. 2011;111(8):1027-32.

40. Cyprowski M, Buczynska A, Kozajda A, Sowiak M, Brodka K, Szadkowska-Stanczyk I. Exposure to (1 -> 3)-beta-D-glucans in swine farms. Aerobiologia. 2012;28(2):161-8.

41. Hong P-Y, Li X, Yang X, Shinkai T, Zhang Y, Wang X, et al. Monitoring airborne biotic contaminants in the indoor environment of pig and poultry confinement buildings. Environmental Microbiology. 2012;14(6):1420-31.

42. Kristiansen A, Saunders AM, Hansen AA, Nielsen PH, Nielsen JL. Community structure of bacteria and fungi in aerosols of a pig confinement building. Fems Microbiology Ecology. 2012;80(2):390-401.

43. Linhares DCL, Cano JP, Wetzell T, Nerem J, Torremorell M, Dee SA. Effect of modified-live porcine reproductive and respiratory syndrome virus (PRRSv) vaccine on the shedding of wild-type virus from an infected population of growing pigs. Vaccine. 2012;30(2):407-13.

44. Sowiak M, Brodka K, Buczynska A, Cyprowski M, Kozajda A, Sobala W, et al. An assessment of potential exposure to bioaerosols among swine farm workers with particular reference to airborne microorganisms in the respirable fraction under various breeding conditions. Aerobiologia. 2012;28(2):121-33.

45. Corzo CA, Culhane M, Dee S, Morrison RB, Torremorell M. Airborne detection and quantification of swine influenza a virus in air samples collected inside, outside and downwind from swine barns. PLoS One. 2013;8(8):e71444.

46. Corzo CA, Romagosa A, Dee SA, Gramer MR, Morrison RB, Torremorell M. Relationship between airborne detection of influenza A virus and the number of infected pigs. Vet J. 2013;196(2):171-5.

47. de Evgrafov MR, Koll P, Frank DN, Baumgartner LK, Robertson CE, Hernandez MT, et al. Molecular Analysis of Bacterial and Circovirus Bioaerosols in Concentrated Animal Feeding Operations. Aerosol Science and Technology. 2013;47(7):755-66.

48. Masclaux FG, Sakwinska O, Charriere N, Semaani E, Oppliger A. Concentration of Airborne Staphylococcus aureus (MRSA and MSSA), Total Bacteria, and Endotoxins in Pig Farms. Annals of Occupational Hygiene. 2013;57(5):550-7.

49. Pavilonis BT, O'Shaughnessy PT, Altmaier R, Metwali N, Thorne PS. Passive monitors to measure hydrogen sulfide near concentrated animal feeding operations. Environ Sci Process Impacts. 2013;15(6):1271-8.

50. Viegas S, Veiga L, Figueredo P, Almeida A, Carolino E, Sabino R, et al. Occupational Exposure to Aflatoxin B-1 in Swine Production and Possible Contamination Sources. Journal of Toxicology and Environmental Health. 2013;76(15):944-51.

51. Yang X, Wang X, Zhang Y, Lee J, Su J, Gates RS. Monitoring total endotoxin and (1 --> 3)-beta-D-glucan at the air exhaust of concentrated animal feeding operations. J Air Waste Manag Assoc. 2013;63(10):1190-8.

52. Agerso Y, Vigre H, Cavaco LM, Josefsen MH. Comparison of air samples, nasal swabs, ear-skin swabs and environmental dust samples for detection of methicillin-resistant Staphylococcus aureus (MRSA) in pig herds. Epidemiol Infect. 2014;142(8):1727-36.

53. Alonso C, Goede DP, Morrison RB, Davies PR, Rovira A, Marthaler DG, et al. Evidence of infectivity of airborne porcine epidemic diarrhea virus and detection of airborne viral RNA at long distances from infected herds. Vet Res. 2014;45:73.

54. Andersen KB, Glasius M, Feilberg A. Gas-particle partitioning of odorants in a pig house measured by thermal desorption GC/MS. Environ Sci Process Impacts. 2014;16(5):1059-68.

55. Bonifait L, Veillette M, Letourneau V, Grenier D, Duchaine C. Detection of Streptococcus suis in Bioaerosols of Swine Confinement Buildings. Applied and Environmental Microbiology. 2014;80(11):3296-304.

56. Brito B, Dee S, Wayne S, Alvarez J, Perez A. Genetic diversity of PRRS virus collected from air samples in four different regions of concentrated swine production during a high incidence season. Viruses. 2014;6(11):4424-36.

57. Corzo CA, Allerson M, Gramer M, Morrison RB, Torremorell M. Detection of airborne influenza a virus in experimentally infected pigs with maternally derived antibodies. Transbound Emerg Dis. 2014;61(1):28-36.

58. Damte D, Yohanes SB, Hossain MA, Lee S-J, Rhee M-H, Kim Y-H, et al. Detection of naturally aerosolized Mycoplasma hyopneumoniae from the air of selected swine farms. Aerobiologia. 2014;30(2):205-9.

59. Kumari P, Choi HL. Seasonal Variability in Airborne Biotic Contaminants in Swine Confinement Buildings. PLoS One. 2014;9(11).

60. Lee S-A, Liao C-H. Size-selective assessment of agricultural workers' personal exposure to airborne fungi and fungal fragments. Science of the Total Environment. 2014;466:725-32.

61. Van Ransbeeck N, Van Langenhove H, Michiels A, Sonck B, Demeyer P. Exposure levels of farmers and veterinarians to particulate matter and gases during operational tasks in pig-fattening houses. Ann Agric Environ Med. 2014;21(3):472-8.

62. Alonso C, Raynor PC, Davies PR, Torremorell M. Concentration, Size Distribution, and Infectivity of Airborne Particles Carrying Swine Viruses. PLoS One. 2015;10(8):e0135675.

63. Arfken AM, Song B, Sung J-S. Comparison of airborne bacterial communities from a hog farm and spray field. Journal of microbiology and biotechnology. 2015;25(5):709-17.

64. Choi MJ, Torremorell M, Bender JB, Smith K, Boxrud D, Ertl JR, et al. Live Animal Markets in Minnesota: A Potential Source for Emergence of Novel Influenza A Viruses and Interspecies Transmission. Clin Infect Dis. 2015;61(9):1355-62.

65. Kumari P, Choi HL. Manure removal system influences the abundance and composition of airborne biotic contaminants in swine confinement buildings. Environmental Monitoring and Assessment. 2015;187(8).

66. Wang CH, Chen BT, Han BC, Liu AC, Hung PC, Chen CY, et al. Field evaluation of personal sampling methods for multiple bioaerosols. PLoS One. 2015;10(3):e0120308.

67. Anderson BD, Ma M, Xia Y, Wang T, Shu B, Lednicky JA, et al. Bioaerosol Sampling in Modern Agriculture: A Novel Approach for Emerging Pathogen Surveillance? J Infect Dis. 2016;214(4):537-45.

68. Ferguson DD, Smith TC, Hanson BM, Wardyn SE, Donham KJ. Detection of Airborne Methicillin-Resistant Staphylococcus aureus Inside and Downwind of a Swine Building, and in Animal Feed: Potential Occupational, Animal Health, and Environmental Implications. J Agromedicine. 2016;21(2):149-53.

69. Neira V, Rabinowitz P, Rendahl A, Paccha B, Gibbs SG, Torremorell M. Characterization of Viral Load, Viability and Persistence of Influenza A Virus in Air and on Surfaces of Swine Production Facilities. PLoS One. 2016;11(1):e0146616.

70. O'Brien KM, Nonnenmann MW. Airborne Influenza A Is Detected in the Personal Breathing Zone of Swine Veterinarians. PLoS One. 2016;11(2):e0149083.

71. Roque K, Lim GD, Jo JH, Shin KM, Song ES, Gautam R, et al. Epizootiological characteristics of viable bacteria and fungi in indoor air from porcine, chicken, or bovine husbandry confinement buildings. J Vet Sci. 2016.

72. Alonso C, Raynor PC, Goyal S, Olson BA, Alba A, Davies PR, et al. Assessment of air sampling methods and size distribution of virus-laden aerosols in outbreaks in swine and poultry farms. J Vet Diagn Invest. 2017:1040638717700221.

73. Walser SM, Brenner B, Wunderlich A, Tuschak C, Huber S, Kolb S, et al. Detection of Legionella-contaminated aerosols in the vicinity of a bio-trickling filter of a breeding sow facility - A pilot study. Sci Total Environ. 2017;575:1197-202.
